# Ginger supplement significantly reduced length of hospital stay in individuals with COVID-19

**DOI:** 10.1186/s12986-022-00717-w

**Published:** 2022-12-28

**Authors:** Yaqi Li, Dawei Yang, Xiwen Gao, Minjie Ju, Hao Fang, Zuoqin Yan, Huanru Qu, Yuanhao Zhang, Linshan Xie, Huifen Weng, Chunxue Bai, Yuanlin Song, Zhirong Sun, Wenye Geng, Xiang Gao

**Affiliations:** 1 Department of Nutrition and Food Hygiene, School of Public Health, Institute of Nutrition, Fudan University, Shanghai, China; 2 Department of Pulmonary and Critical Care Medicine, Zhongshan Hospital, Fudan University, Shanghai, China; 3 Shanghai Engineer and Technology Research Center of Internet of Things for Respiratory Medicine, Shanghai, China; 4 Department of Pulmonary and Critical Care Medicine, Minhang Hospital, Fudan University, Shanghai, China; 5 Department of Critical Care Medicine, Zhongshan Hospital, Fudan University, Shanghai, China; 6 Department of Anesthesiology, Zhongshan Hospital, Fudan University, Shanghai, China; 7 Department of Anesthesiology, Minhang Hospital, Fudan University, Shanghai, China; 8 Zhongshan Hospital, Fudan University, Shanghai, China; 9 Department of Rheumatology, Longhua Hospital Affiliated to Shanghai University of Traditional Chinese Medicine, Shanghai, China; 10 Huashan Hospital, Fudan University, Shanghai, China; 11 Shanghai Suvalue Healthcare Scientific Co., Ltd., Shanghai, China; 12 Shanghai Key Laboratory of Lung Inflammation and Injury, Shanghai Institute of Infectious Disease and Biosecurity, Shanghai, China; 13 Department of Anesthesiology, Fudan University Shanghai Cancer Center, Shanghai, China; 14 Department of Oncology, Shanghai Medical College, Fudan University, Shanghai, China; 15 Fudan Zhangjiang Institute, Fudan University, Shanghai, China

**Keywords:** COVID-19, Hospital length of stay, Ginger, Randomized control trial

## Abstract

**Background:**

Evidence from previous studies has suggested that ginger extract exhibits the potential as an alternative treatment for Coronavirus disease 2019 (COVID-19). Here, we want to investigate whether ginger supplement improves the clinical manifestation of hospitalized COVID-19 individuals.

**Methods:**

A total of 227 hospitalized individuals with COVID-19 were randomized to either the control (*n* = 132) or intervention group (*n* = 95). The intervention group took ginger supplement orally at the dosage of 1.5 g twice daily, until they were discharged from the hospital. Both groups received the same standard of general medical care during hospitalization, and the length of stay was recorded and compared between groups.

**Results:**

Among all participants, a significant reduction in hospitalization time (the difference between the treatment and control groups was 2.4 d, 95% CI 1.6–3.2) was detected in response to the ginger supplement. This effect was more pronounced in men, participants aged 60 years or older, and participants with pre-existing medical conditions, relative to their counterparts (*P-interactions* < 0.05 for all).

**Conclusion:**

Ginger supplement significantly shortened the length of stay of hospitalized individuals with COVID-19.

**Trial registration:** The trial was registered on the Chinese Clinical Trial Registry (ChiCTR2200059824).

## Background

Coronavirus disease 2019 (COVID-19) is an infectious disease induced by the novel severe acute respiratory syndrome coronavirus 2 (SARS-CoV-2). As one of the largest pandemics in recent centuries, as of August 2022, COVID-19 has caused more than 590 million cases, including over 6 million deaths; continuously challenging the global health system, increasing medical burdens and severely threatening people’s lives. While immunization with vaccines proved the efficacy of protecting people from virus infection [1, 2], with the emerging new strains of the virus and decay of antibodies after vaccination [3, 4], the risk of infection could increase [5]. Therefore, finding effective interventions to help people recover from COVID-19 is of equal importance as building up immunity via vaccination.

Ginger (*Zingiber officinale*), as an edible plant, is also considered as a traditional herbal medicine in China. Scientific evidence has demonstrated that ginger has broad anti-inflammatory properties through modulating cytokine production and other regulatory pathways [6–9]. Specifically, ginger exhibited a protective role against acute respiratory distress syndrome [10, 11], the primary cause of mortality in severe COVID-19 cases. Moreover, bioactive compounds present in ginger, such as gingerol and shogaol, showed high affinity to the SARS-CoV-2 spike protein, thus could potentially interfere with the spike protein and host angiotensin-converting enzyme 2 (ACE-2) interaction [12, 13]. Interactions between ginger-derived compounds and papain-like protease and other virus proteins essential for its survival were also indicated via computational theoretical models [14, 15].

Taken together, ginger presents the potential as an alternative therapeutic agent to treat COVID-19. Therefore, we conducted a clinical trial to examine the effect of the ginger supplement on the clinical manifestations of individuals with COVID-19.

## Methods

### Study design and participants

The study protocol was approved by the institutional review boards of Zhongshan Hospital, Fudan University, and the trial was registered on the Chinese Clinical Trial Registry (ChiCTR2200059824). All participants provided written informed consent. Study participants were recruited among the individuals with COVID-19 hospitalized at two Fangcang shelter hospitals, from April to May 2022, in Shanghai, China. At the time of admission, individuals with COVID-19 tested positive but no symptoms (asymptomatic infection) were invited to participate in the study.

### Sample-size estimation

As our study is experimental in nature with no prior reference to estimate the sample size for treatment effect testing, we made our best effort to recruit eligible participants and obtain an acceptable sample size.

### Randomization and masking

Eligible participants were randomized to either the ginger supplement group or the control group, and no masking was implemented in the current trial.

### Intervention

The ginger supplements [Fujian Longzhi Biotechnology Co., Ltd (Fujian, China)] were provided in the form of ginger powder packed individually (1.5 g/bag), participants in the ginger supplement group were asked to mix it with hot water and take it orally at the dosage one bag per time twice daily (before breakfast and lunch), the intervention continued until the participants were discharged from the hospital. The standard for hospital discharge was their throat swab test for COVID-19 reached 35 (CT value) for consecutive 2 days without major symptoms, including but not limited to sore throat, stuffy nose, fever, and cough. Both groups received the same general medical care during the hospitalization period.

### Outcome

The primary outcome was the length of stay before participants were recovered and discharged from hospitals, where they had repeated negative results of COVID-19 tests and were cleared of COVID-19 as judged by the physicians in charge.

### Statistical analyses

Descriptive statistics are presented as means ± SDs or numbers (percentages) for numeric and categorical variables, respectively. Baseline characteristics between groups were compared using t-test or χ^2^ tests when appropriate. Generalized linear models were used to analyze the effect of ginger supplement adjusted for age, sex, location, chronic disease status (hypertension, diabetes, other chronic diseases), and surgical history. To examine whether the potential effects of the ginger supplement on hospitalization time were modified by other factors, we tested the interaction between sex and intervention, age and intervention, and pre-existing medical conditions (combined data of diseases status and surgical history) and intervention by adding the interaction component in our model, adjusting for aforementioned covariates. Subgroup analyses were subsequently conducted when the interaction terms were significant. All analyses were performed using SAS version 9.4 (SAS Institute Inc), and a 2-sided *P* value < 0.05 was considered statistically significant.

### Role of funding source

The funding source did not play any role in study design, data collection, analyses, manuscript preparation, or decision to submit the work for publication.

## Results

From April to May 2022, a total of 254 participants were recruited from two Fangcang shelter hospitals, and 227 completed the intervention. The baseline characteristics of these participants are shown in Table 1. Except for a relatively higher proportion of individuals with hypertension in the control group (*P* = 0.05), the sex and age distribution and other basic health conditions were the similar between two groups.

**Table 1:** Baseline Participants Characterristics

Characteristics	Control group (n = 132)	Ginger treatment group (n = 95)
Men, no. (%)	85 (64)	62 (65)
Age, y	56 ± 17	53 ± 13
Hypertension, no. (%)	39 (30)	17 (18)*
Diabetes, no. (%)	17 (13)	7 (7)
Other chronic diseases, no. (%)	25 (19)	11 (12)
Surgical history, no. (%)	9 (7)	2 (2)

Data are presented as means ± SDs or numbers (percentages)

*Indicates statistical significance, *P* = 0.05

The ginger-supplemented group showed a significantly shorter hospitalization time compared with the control (6.0 vs. 8.5 d, *P* < 0.0001), with an estimated difference of 2.4 d [95% CI 1.6–3.2 d] (Table 2).

**Table 2:** Primary and secondary outcomes for control and ginger-supplemented intervention group

Outcomes	Model estimated	Mean (95% CI)	Intervention difference estimate (95% CI)	*P* value
	Control	Ginger		
*Primary outcome*				
Participants, no (%)	132 (58)	95 (42)		
Unadjusted means, d	8.5 (8.0–9.0)	6.1 (5.5–6.7)	2.4 (1.6–3.2)	< 0.0001
Adjusted means, d	8.5 (7.9–9.0)	6.0 (5.4–6.7)	2.4 (1.6–3.2)	< 0.0001
By sex^1^				
*Men (n = 147)*				
Length of stay, d	8.9 (8.2–9.5)	5.8 (5.0–6.6)	3.0 (2.1–4.0)	< 0.0001
*Women (n = 80)*				
Length of stay, d	7.8 (6.9–8.8)	6.7 (5.5–7.8)	1.2 (-0.26–2.6)	0.11
By Age^2^				
*Age < 60 (n = 143)*				
Length of stay, d	7.8 (7.1–8.5)	6.6 (5.9–7.4)	1.2 (0.28–2.1)	0.010
*Age ≥ 60 (n = 84)*				
Length of stay, d	9.9 (9.0–10.7)	4.9 (3.8–6.1)	4.9 (3.5–6.4)	< 0.0001
By prior medical conditions^3^				
*W/prior medical conditions (n = 87)*				
Length of stay, d	9.6 (8.9–10.4)	5.8 (4.7–6.9)	3.8 (2.5–5.2)	< 0.0001
*W/O prior medical conditions (n = 140)*				
Length of stay, d	7.9 (7.1–8.6)	6.1 (5.3–6.8)	1.8 (0.80–2.8)	0.0005

Results were adjusted for sex, age, and prior medical conditions, including hypertension, diabetes, other chronic diseases, and surgical history, as needed

^1^*P*-interaction was 0.04 for sex, 0.004 for age, and 0.035 for the status of prior medical conditions

The effects of the ginger supplement on COVID-19 appeared to be modified by sex, age, and prior medical status (P-interaction < 0.05 for all; Table 2). While ginger supplement significantly shortened the length of stay by 3 days [95% CI 2.1–4.0 d] in men (5.8 vs. 8.9 d, *P* < 0.0001), this beneficial effect was not observed in women (6.7 vs. 7.8 d, *P* = 0.11). Further, when categorized by age groups, compared to their counterparts, both age groups benefited from the ginger supplement; especially for the participants aged 60 years or older, an almost 5-day [4.9 d, 95% CI 3.5–6.4 d] reduction in hospitalization time was estimated (*P* < 0.0001). Additionally, when classified by prior medical conditions, ginger-supplemented participants showed significant improvement in hospital stays regardless of their prior health status; where the estimated difference was 3.8 days [95% CI 2.5–5.2 d] in individuals with prior medical conditions and 1.8 days [95% CI 0.8–2.8 d] in participants without pre-existing medical conditions, respectively.

## Discussion

To our knowledge, this is the first clinical trial focusing on the effect of the ginger supplement on the clinical manifestation, evaluated as the length of hospital stays, of hospitalized individuals with COVID-19. A significant improvement in hospitalization time was observed in response to the ginger supplement. One possible explanation for the observed beneficial effect of the ginger supplement on the length of stay in study participants could be the ginger-derived exosomal micro-RNA, which has been demonstrated to inhibit lung inflammation caused by COVID-19 in both in vitro and in vivo studies [16].

Interestingly, when stratified by sex, only men benefited from the ginger supplement, whereas no intervention effect was detected in women. This result aligned with the observed differences in manifestation and severity of COVID-19 between men and women reported in previous studies [17, 18], while the underlying mechanism regarding the sex differences in immunopathogenesis needs further investigation, these studies implied a possible difference in the host immunity related to sex and suggesting the necessity of considering potential sex effect in future clinical studies. Besides, our subgroup analysis highlighted the importance of ginger supplement to older adults, where the hospitalization time could be shortened to less than half of their un-supplemented comparisons, which could effectively reduce the suffering and pain and improve the life quality of the elderly. Aging has been recognized as a critical risk factor for the severity and mortality of COVID-19 [19, 20], possibly due to age-related general decline in immune function. Therefore, the beneficial effect of the ginger supplement on the length of hospital stay could be less prominent in younger adults given their relatively quicker recovery compared to the elderly. Last but not the least, we explored the potential impact of pre-existing medical conditions when infected with COVID-19. While the participants with prior health issues tended to have longer hospital stays, supplementing with ginger significantly shortened the length of stay by about 40%, suggesting a strong protective effect of ginger supplement to this vulnerable group.

This study has several limitations. The primary outcome we focused on here is the length of stay in the hospital, it would be better to record other clinical symptoms of COVID-19 to evaluate the potential impact of ginger supplementation on other aspects of the clinical manifestation of COVID-19. Thus this study should be considered preliminary. Further, biological samples (such as blood) were not collected in the current study, and we cannot link the observed difference in hospitalization time to possible changes in the immune response. The future study thus could examine the circulating level of inflammatory biomarkers, and further investigate the underlying mechanism of the observed beneficial effect of the ginger supplement.

In conclusion, this preliminary study suggested that supplementing individuals with COVID-19 with ginger could be a cost-effective strategy in the future treatment of COVID-19, and emphasized the importance of implementing ginger supplementation to the target population to achieve the best outcomes.

## Data Availability

The data that support the findings of this study are available from the corresponding authors upon reasonable request.

## Abbreviations

COVID-19: Coronavirus disease 2019
ACE-2: Angiotensin-converting enzyme 2
